# The role of cerebrospinal fluid cross-section area ratio in the prediction of dural ossification and clinical outcomes in patients with thoracic ossification of ligamentum flavum

**DOI:** 10.1186/s12891-021-04574-1

**Published:** 2021-08-17

**Authors:** Jiliang Zhai, Shigong Guo, Yu Zhao, Chunxu Li, Tong Niu

**Affiliations:** 1 Department of Orthopaedic Surgery, Peking Union Medical College Hospital, Chinese Academy of Medical Sciences and Peking Union Medical College, shuaifuyuan 1#, Dongcheng district, Beijing, China; 2 Department of Rehabilitation Medicine, Southmead Hospital, Bristol, UK

**Keywords:** Ossification of the ligamentum flavum, Dural ossification, Cerebrospinal fluid leakage, Cross-sectional area, Cerebrospinal fluid cross-section area ratio

## Abstract

**Background:**

It is imperative to preoperatively distinguish dural ossification (DO) and thus anticipate the risks and outcome of the surgery for patients with ossification of ligamentum flavum (OLF). However, studies have disagreed as to the efficacy of the radiographic signs or factors to predict DO and surgical outcome. In additon, the association between the cerebrospinal fluid cross-section area ratio (CCAR) and DO or clinical outcome had not been reported. The purpose of this study was to analyse CCAR and its role in prediction of DO and neurological function recovery rate in patients with OLF.

**Methods:**

Fifty-two consecutive patients with OLF, who underwent posterior thoracic decompression and fusion between September 2012 and March 2019 at a single institution, were retrospectively reviewed. Demographic data, radiographic signs of DO, CCAR, pre- and postoperative modified Japanese Orthopedic Association (mJOA) score were recorded.

**Results:**

There were 27 patients in the DO group and 25 patients in the non-DO group, with a mean age at surgery of 57.4 years and 53.9 years, respectively. No significant differences were found in sex, age, segment of maximum compression and preoperative mJOA score between the two groups. The receiver operating characteristic curve showed that the value of CCAR had a relatively high value for diagnosis of DO and prediction of neurological function recovery rate (*P* = .000). According to the value of CCAR, three zones were defined as DO zone (≤14.3%), non-DO zone (≥44.5%), and gray zone (14.3 to 44.5%). When the value of CCAR≤14.3%, the recovery rate was poor or fair, while it had good or excellent recovery when CCAR≥45.2%.

**Conclusion:**

The value of CCAR had a high diagnostic value for prediction of DO and neurological function recovery rate in patients with OLF.

## Background

Ossification of the ligamentum flavum (OLF) is the major cause of thoracic myelopathy and has been frequently reported in Japan and other East Asian countries. The incidence of OLF ranges from 3.8 to 64% as reported in previous literatures [1–10]. Surgery is the best method to treat patients with OLF and neurological dysfunction [11, 12]. However, the incidence of the most common complication, namely cerebrospinal fluid (CSF) leakage, is high during surgery and poses a difficult problem for surgeons to overcome [13]. The main reason for CSF leakage is dural ossification (DO), with a incidence ranged from 11 to 66.6% [3, 4, 10, 14–20].

Preoperative identification of DO and prediction of surgical outcome are very important for surgeons to adopt an appropriate surgical strategy and manage dural tear during surgery [3, 15, 21]. It is also helpful for surgeons to counsel patients about the risks of surgery [10]. However, radiographic study about the signs of DO and neurological function have rarely been reported in the literature due to the low incidence of OLF and DO [3, 6, 10, 16, 22, 23]. and the diagnostic accuracy still needs to be improved [10, 15, 23]. In our opinion, cross-section area (CSA) of cerebrospinal fluid indicates the compensatory space for spinal cord. A patient with large CSA of ossification mass may present without DO and with mild neural dysfunction damage, when the compensatory space of cerebrospinal fluid is large, and vice versa.

The aim of this retrospective study was to (1) measure the value of cerebrospinal fluid cross-section area ratio (CCAR) on magnetic resonance imaging (MRI) and computed tomography (CT) images; (2) investigate the role of CCAR in prediction of DO and neurological function recovery rate.

## Materials and methods

### Patients

The patients of OLF with thoracic myelopathy, who underwent posterior thoracic laminectomy and fusion with instrumentation between September 2012 and March 2019 at a single institution, were retrospectively reviewed. The study was conducted in accordance with the Declaration of Helsinki and informed consent was waived, which was approved by the Ethical Committee of Peking union medical college hospital. No patients participated in this study and private information was protected. The exclusion criteria were patients with diffuse idiopathic skeletal hyperostosis and concurrent thoracic ventral compressive lesions, such as thoracic disc herniation with protrusion or extrusion, thoracic kyphosis and ossification of the posterior longitudinal ligament, previous history of thoracic surgery, thoracic trauma, infection and tumor.

All patients had both CT and MRI images. CT had been performed on a dual-energy CT scanner, Discovery CT1750 (GE Healthcare) or Somatom Definition Flash (Siemens Healthineers). MRI had been performed on a 3-T scanner, Discovery MR1750 (GE Healthcare) or Magnetom Skyra (Siemens Healthineers). Intraoperative features of DO included an ossified dura that was fused with OLF, the inability to resect the OLF from the ossified portion of the dura mater during surgery [4, 17] and obvious ossified dura in the resected ossified mass [13, 23]. Ossified dura was resected if DO was present and “floating technique” was not adopted in this study. The dural laceration was repaired with onlay technique with gelatin sponge, artificial dura or autologous fascia. Watertight suturing was performed layer by layer and a subfascial drainage was placed.

Demographic data, Sato classification of OLF, radiographic signs of DO, including tram track sign, comma sign and bridge sign, pre- and postoperative neurological function were recorded. The modified Japanese Orthopedic Association (mJOA) scoring system for thoracic myelopathy (11 point scale) was used to evaluate pre- and postoperative neurological status. The recovery rate (RR) was calculated by the following formula: RR = (mJOA score at last follow-up – preoperative mJOA score)/ (11 - preoperative mJOA score) × 100% [5]. A rate of 75–100% was graded as excellent, 50–74% as good, 25–49% as fair, and 0–24% as poor.

### Radiographic measurement

Image J software (National Institutes of Health, Bethesda, MD, USA) was used for radiographic measurement. To ensure reliability, imaging evaluation was performed before the review of medical records and two independent observers were responsible for the measurement. All images were measured twice by each observer, and the mean of the two measurements was considered for statistical analysis.

**Fig. 1:**
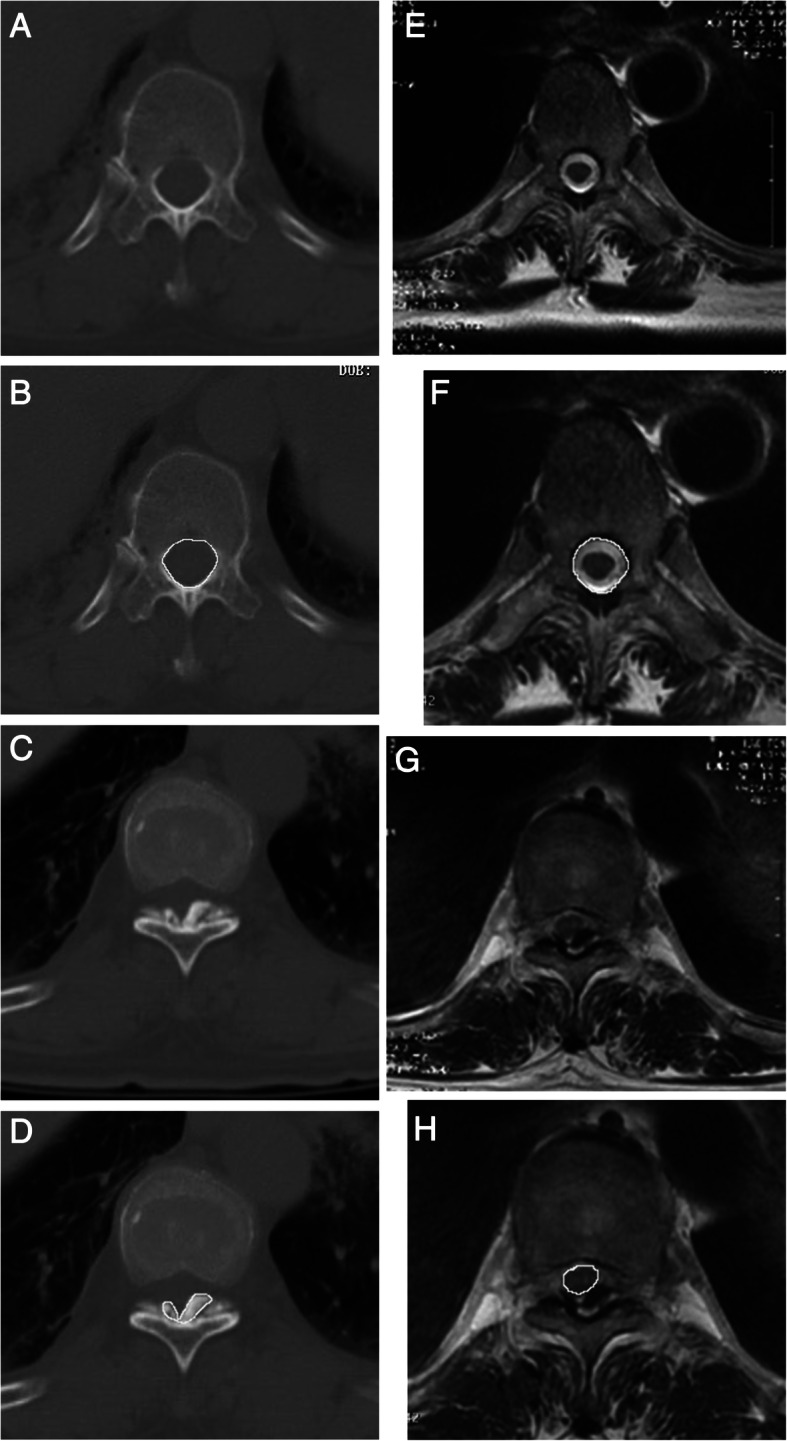
Radiographic measurement. (**A)** Normal spinal canal at the pedicle level of the maximum compression segment. (**B)** Cross-section area (CSA) of the normal spinal canal. **(C)** Axial view of computed tomography scan at the segment of maximum compression. **(D)** CSA of the ossified mass. **(E)** Magnetic resonance imaging (MRI) at the pedicle level. **(F)** Measurement of CSA of the dural sac at the pedicle level. **(G)** MRI at the segment of maximum compression. **(H)** CSA of the spinal cord

It is inaccurate to measured CSA of cerebrospinal fluid directly on MRI, because the demarcation between dura mater and ossification mass on MRI is not clear. Therefore, we calculated the CSA ratio of cerebrospinal fluid to spinal canal indirectly by measuring the CSA ratio of ossification mass to spinal canal on CT and that of spinal cord to dural sac on MRI, respectively. CSA of normal spinal canal on CT image was measured at the pedicle region (usually without ossification) near the narrowest segment, where the distance between the pedicles was the widest (Fig. 1A). The average value of CSA of the dural sac of adjacent upper and lower segment was supposed to be equal to that of the normal spinal canal. The axial CT and MRI images at the maximum compression level were chosen for measurement of CSA of ossified mass and spinal cord, because DO mostly occurred solely in the segment with maximum compression [11]. First, we made a calibration for Image J software according to the scale on the axial CT image. Then, we drew a circle along the bone vertebral canal border, and the Image J software automatically calculated the CSA at the pedicle section (Fig. 1B). CSA of the ossified mass was measured in the same way by drawing a circle around the mass on the CT image (Fig. 1C and D). Next, the CSA of the dural sac at the adjacent upper and lower segments were measured with a circle drawn along the border of the dural sac on MRI image (Fig. 1E and F). Lastly, we measured the CSA of the spinal cord on the MRI image at the same narrowest segment (Fig. 1G and H). The value of ossified mass cross-section area ratio (OCAR), spinal cord cross-section area ratio (SCAR) and CCAR were expressed as follows:

$$ OCAR=\frac{\mathrm{CSA}\ \mathrm{of}\ \mathrm{the}\ \mathrm{ossified}\ \mathrm{mass}}{\mathrm{CSA}\ \mathrm{of}\ \mathrm{the}\ \mathrm{normal}\ \mathrm{spinal}\ \mathrm{canal}}\times 100\% $$

$$ \mathrm{SCAR}=\frac{\mathrm{CSA}\ \mathrm{of}\ \mathrm{the}\ \mathrm{spinal}\ \mathrm{cord}\ \mathrm{at}\ \mathrm{the}\ \mathrm{narrowest}\ \mathrm{level}}{\mathrm{average}\ \mathrm{CSA}\ \mathrm{of}\ \mathrm{dural}\ \mathrm{sac}\ \mathrm{of}\ \left(\mathrm{adjacent}\ \mathrm{upper}\ \mathrm{segment}+\mathrm{lower}\ \mathrm{segment}\right)}\times 100\% $$

$$ \mathrm{CCAR}=\left(1-\mathrm{OCAR}-\mathrm{SCAR}\right)\times 100\% $$

### Statistical analysis

The Statistical Package for Social Sciences (SPSS Inc., Chicago, IL, USA) software was used for data analysis. Interclass correlation coefficients (ICCs) were used to assess interobserver reliability. t-test was used for continuous variables, whereas the chi-square test was used for categorical variables. Pearson’s and Spearman’s correlation coefficients were used to analyze the association between CCAR and DO or RR. Receiver operating characteristic (ROC) curve was drew and area under the curve (AUC) value was used to determine the best cut-off value of the parameters for diagnosing DO and prognosticating RR. A *P* value < 0.05 was considered statistically significant.

## Results

Fifty-two consecutive patients (28 female and 24 male) were ultimately included in the study sample. The duration of symptoms varied from 2 months to 28 years (5.3 ± 6.2 years) before visiting the hospital. The mean age at surgery was 55.6 ± 9.4 years (30–74), with a mean follow-up time of 4.6 ± 2.2 years (0.6–7.2). There were 27 patients in the DO group and 25 in the non-DO group, with a follow-up of 4.5 ± 2.1 years and 4.6 ± 2.3 years, respectively. The most commonly affected segment of DO was T9–12, with an incidence of 34.6% (18/52).

No significant differences were found in sex, age, segment of maximum compression and preoperative mJOA score between the DO group and non-DO group (Table 1). In the DO group, the most common morphologic configurations according to the Sato classification were fused type and tuberous type, while lateral type and extended type were the major types in the non-DO group. All patients with OLF of tuberous type, tram track sign (+) or comma sign (+) had DO. Additonally, most of the patients (6/7) with bridge sign (+) also had DO (Table 2). The ICC value for both observers who were responsible for the radiographic measurement was 0.947 (95% confidence interval [CI], 0.909 to 0.969), which suggests high inter-observer agreement.

**Table 1:** Patients’ demographic characteristics

	Non-DO group	DO group	*P* value
No. of patients	25	27	/
Sex (male/female)	14/11	10/17	/
Age (yr)	57.4 ± 9.2	53.9 ± 9.6	0.191
Follow-up time (yr)	4.5 ± 2.1	4.6 ± 2.3	0.793
Segment of maximum compression			
T1-T4	4	4	/
T5-T8	3	5	/
T9-T12	18	18	/
Neurological function			
Pre-op mJOA score	5.7 ± 2.2	5.1 ± 3.0	0.409
FU mJOA score	9.4 ± 1.5	7.4 ± 2.9	0.004
RR (%)	72.5 ± 23.0	42.3 ± 32.5	0.000

*Abbreviations*: *DO* dural ossification, *Pre-op mJOA score* preoperative mJOA score, *FU mJOA score* last follow-up mJOA score, *RR* recovery rate

**Table 2:** Radiographic features and measurement results

	Non-DO group	DO group	*P* value
Sato classification			
Lateral type	12	0	/
Extended type	5	4	/
Enlarged type	4	2	/
Fused type	4	5	/
Tuberous type	0	16	/
TTS (+)	0	8	0.004
CS (+)	0	9	0.002
BS (+)	1	6	0.101
CCAR (95% CI)	50.0% (45.1–54.8%)	22.2% (17.4–27.0%)	0.000

*Abbreviations*: *DO* dural ossification, *TTS* tram track sign, *CS* comma sign, *BS* bridge sign, *CCAR* CSF cross-section area ratio

**Fig. 2:**
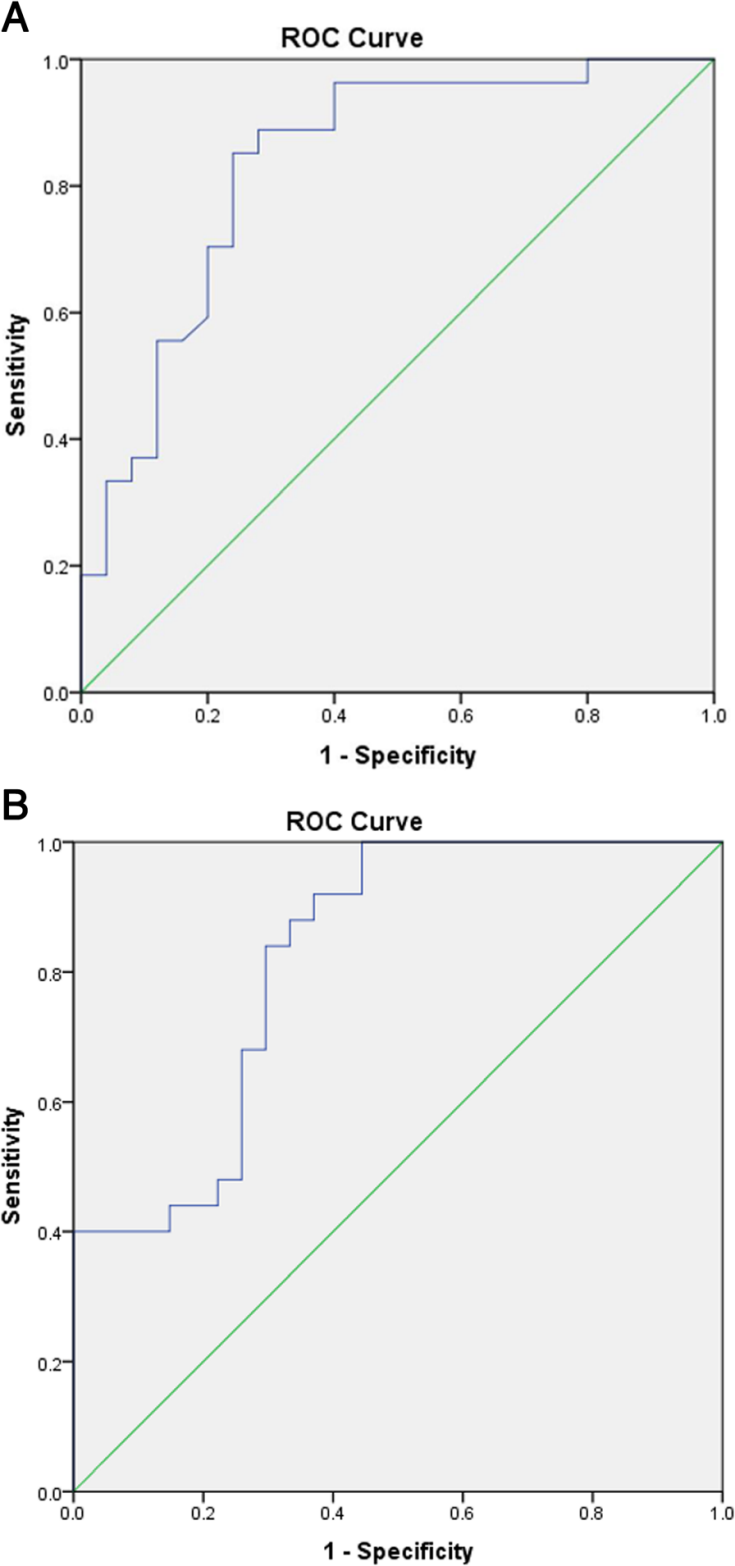
Receiver operating characteristic (ROC) curve. (A) The value of CSF cross-section area ratio (CCAR) has a high diagnostic value for dural ossification (area under the curve [AUC] = 0.835 [95% confidence interval [CI], 0.722 to 0.948]; *P* < 0.001). (B) The value of CCAR has a relatively high prognosis value for postoperative neurological function recovery rate (AUC, 0.822 [95% CI, 0.709 to 0.935]; *P* < 0.001)

The value of CCAR in the group with DO was significantly lower than that in the group without DO (*P* = 0.000) (Table 2). The ROC curve showed that CCAR had a relatively high diagnostic value for DO (AUC = 0.835 [95% CI, 0.722 to 0.948]; *P* = 0.000), and the cut-off value was 36.4% (Fig. 2A). The sensitivity and specificity were 87.0% and 82.8%, respectively. All patients suffered from DO when the value of CCAR was equal to or less than 14.3% and DO zone was defined as CCAR≤14.3%. Similar to this, the non-DO zone was designated as CCAR≥44.5%, and the gray zone was defined when the value of CCAR was between 14.3 and 44.5% (Table 3).

**Table 3:** The analysis of CCAR for prognosis of dural ossification

CCAR	Sensitivity	Specificity	Diagnostic Coincidence Rate
< 14.3%	59.5% (43.3 to 74.4%)	100% (69.2 to 100%)	67.3%
< 36.4%	87.0% (66.4 to 97.2%)	82.8% (64.2 to 94.2%)	84.6%
< 44.5%	100% (79.4 to 100%)	75.0% (57.8 to 87.9%)	80.8%

*Abbreviation*: *CCAR* CSF cross-section area ratio

One case in non-DO group and two cases in DO group suffered from muscle weakness postoperatively. The mJOA score and recovery rate at the last follow-up were significantly higher in the non-DO group. The optimal cut-off value of CCAR to predict neurological function recovery rate was 42.7% (AUC = 0.8 22[95% CI, 0.709 to 0.935]; *P =* 0.000), with a sensitivity and specificity of 89.5% and 69.7%, respectively (Fig. 2B). When the value of CCAR≤14.3%, the recovery rate was poor or fair, while the recovery rate was good or excellent when the value≥45.2% (Table 4).

**Table 4:** The analysis of CCAR for prediction of neurological function recovery rate

CCAR	Sensitivity	Specificity	Diagnostic Coincidence Rate
< 14.3%	64.3% (48.0 to 78.4%)	100% (69.2 to 100%)	71.2%
< 42.7%	89.5% (66.9 to 98.7%)	69.7% (51.3 to 84.4%)	76.9%
< 45.2%	100% (78.2 to 100%)	67.6% (50.2 to 82.0%)	76.9%

*Abbreviation*: *CCAR* CSF cross-section area ratio

## Discussion

DO is an uncommon but troublesome disease in patients with OLF. Preoperative diagnosis of DO enables the surgeon to make adequate preparation and improve the safety of the surgery [10]. However, it is difficult for surgeons to confirm the diagnosis of DO preoperatively based on clinical and radiographic examination [16].

The rate of DO in tuberous type of Sato classification was much higher than other types [15]. Muthukumar [10] reported that tram track sign (TTS) and comma sign (CS) could be used to predict DO in patients with OLF. However, in the study by Sun et al. [15], the diagnostic specificity of TTS was only 59%. Li et al. [3] proposed a new characteristic imaging sign, namely ‘bridge sign’ (BS). The authors reported that all seven patients with BS in this study had DO [3]. ‘T2 ring sign’ on MRI also had a high correlation with DO. However, there was a total of only 10 cases of DO in this study [24].

Zhou et al. [16] found that the risk of DO increased significantly when unilateral spinal canal occupational rate was at least 60%. In addition, they proposed a grading system and DO was highly suspected when a score > 2 [16]. However, unilateral measurement could not accurately reflect the degree of bilateral compression. In our previous study, we measured the occupational rate of ossified mass and recommended a value of CSA occupying ratio < 45% as the safe zone, and > 55% as the ossification zone in the T9-T12 subgroup [23].

Mechanical stress between the OLF and dura mater play an important role in the onset of DO [25–27] and was related with CSA of cerebrospinal fluid. Therefore, we speculate that CCAR may be another important factor in predicting DO. In this study, the optimal cut-off value of CCAR for prediction of DO was 36.4%. DO zone (≤14.3%), non-DO zone (≥44.5%) and gray zone (14.3 to 44.5%) was defined according to the value of CCAR. Thus, surgeons should highly suspect the existence of DO when the value of CCAR is less than 14.3%.

The neurological recovery rate was another concern and ranged from 31 to 100% after surgery for patients with OLF [20, 24, 28, 29]. Various factors have been reported to be associated with surgical outcome [6, 13, 14, 24, 30, 31] and the results were inconsistent among different studies [32]. Age at surgery, gender, level of the ossified lesion, number of segments of OLF, coexisting ossification of the posterior longitudinal ligament, OLF type, and intramedullary signal intensity did not predict postoperative recovery in several studies [30, 33, 34]. Preoperative duration of symptoms, preoperative JOA score and the degree of compression have been considered the most consistent factors in most of the studies [24, 30, 33, 35, 36]. It has been reported that the spinal canal diameter (paramedian and sagittal) and CSA occupying ratio were the top three relevant continuous variables with thoracic myelopathy. The optimal cut-off value for the diagnosis of OLF-induced myelopathy were 60, 50 and 80%, respectively [37]. Lee et al. [38] measured six radiographic parameters on CT and showed that the area of OLF ratio was found to be the best radiological parameter and was highly correlated with OLF-induced thoracic myelopathy. Sanghvi et al. [39] also found that the preoperative dural canal grade significantly correlated with the neurological recovery rate in OLF cases.

However, the measurement results were not always consistent with the neural function. In a retrospective case-control study, 19 patients with moderately compression (Grade III) had thoracic myelopathy, while 28 moderately compressed patients had no neurological symptoms [37]. The reason for the inconsistent results may be due to different static pressures on the spinal cord caused by different CSA of CSF [38]. Thus, it may be assumed that the grade of CCAR was correlated with the rate of neurological recovery rate. In this study, we found that the neurological recovery rate was highly correlated with the value of CCAR. A value of CCAR≤14.3% predicted poor or fair recovery rate of mJOA score, while CCAR≥45.2% meant good or excellent recovery rate. Therefore, it is better to perform the surgery when the value of CCAR is more than 45.2% to achieve better neurological recovery.

This study had some limitations. First, the sample size was relatively small. Nevertheless, it is difficult to adopt large number of cases in one single center due to the low incidence of thoracic OLF and strict inclusion criteria. Therefore, further multicenter studies with large numbers of patients are necessary to verify these preliminary findings. Second, there is concern of possible selection bias due to retrospective study design. However, all patients of OLF were reviewed and only patients who met our criteria were enrolled. Third, there might have errors associated with radiographic measurement on MRI or CT image due to differences in cutting angles induced by technical problems. However, radiologists were well trained to follow the same guidelines when performing CT or MRI scans and the cutting angle for the same segment was the same.

## Conclusions

This study showed that CCAR had a close relationship with DO as well as the recovery rate of neurological function. A value of CCAR≤14.3% indicated the presence of DO and poor neurological outcome. To achieve better neurological function recovery, surgical decompression is recommended when the value of CCAR≥45.2%.

## Data Availability

The datasets used and/or analysed during the current study are available from the corresponding author on reasonable request.

## Abbreviations

OLF: Ossification of ligamentum flavum
DO: Dural ossification
CCAR: Cerebrospinal fluid cross-section area ratio
CSF: Cerebrospinal fluid
CSA: Cross-section area
MRI: Magnetic resonance imaging
CT: Computed tomography
mJOA: modified Japanese Orthopedic Association
RR: Recovery rate
OCAR: Ossified mass cross-section area ratio
SCAR: Spinal cord cross-section area ratio
ICCs: Interclass correlation coefficients
ROC: Receiver operating characteristic
CI: Confidence interval
AUC: Area under the curve
TTS: Tram track sign
CS: Comma sign
BS: Bridge sign
